# Advancements in the application of precision nursing model on hemodialysis for diabetic nephropathy: A review

**DOI:** 10.1097/MD.0000000000040952

**Published:** 2024-12-20

**Authors:** Jing Ma, Dan-Wei Ma

**Affiliations:** a Department of Nursing Care, The Second Affiliated Hospital of Mudanjiang Medical University, Mudanjiang, China; b Medical Section, The Second Affiliated Hospital of Mudanjiang Medical University, Mudanjiang, China.

**Keywords:** clinical nursing, diabetic nephropathy, health management, hemodialysis, precision nursing model

## Abstract

This study explores the application and advancements of precision nursing model (PNM) in hemodialysis for diabetic nephropathy patients. Diabetic nephropathy is a severe complication of diabetes, frequently leading to end-stage renal disease and necessitating long-term hemodialysis. The PNM aims to enhance treatment outcomes and patient quality of life through individualized care plans, multidisciplinary collaboration, and continuous quality improvement. Research indicates that this model significantly improves clinical indicators and patient satisfaction, demonstrating broad applicability. This paper provides a detailed overview of the definition, theoretical foundation, implementation strategies, and specific interventions of the PNM, and evaluates its effectiveness in hemodialysis. Additionally, it addresses the challenges faced in implementation and suggests future research directions. Emphasis is placed on the need for long-term studies, technological innovations, and cost-effectiveness analyses to further integrate precision nursing into clinical practice.

## 1. Introduction

### 1.1. Epidemiology and implications of diabetic nephropathy (DN)

DN is one of the most common and severe complications of diabetes, with its incidence and mortality rates increasing globally.^[1]^ Approximately 20% to 50% of diabetic patients are estimated to develop DN, with this proportion being even higher in certain high-risk populations.^[2]^ DN has become a leading cause of end-stage renal disease (ESRD), significantly elevating the risk of cardiovascular events and mortality in affected individuals.^[3]^ The pathological features of DN include thickening of the glomerular basement membrane, mesangial matrix expansion, and glomerulosclerosis, ultimately leading to irreversible kidney function impairment.^[3]^ Thus, DN not only severely impacts patients’ quality of life but also imposes a substantial economic burden on healthcare systems and society.

### 1.2. Current status of hemodialysis as a primary treatment for DN

Hemodialysis is a primary renal replacement therapy for patients with end-stage DN.^[4]^ It involves using a machine to remove toxins and excess fluids from the blood, substituting for the lost kidney function. While hemodialysis can prolong the lives of DN patients, it is associated with various complications and challenges, such as vascular access issues, inadequate dialysis, hypotension, and electrolyte imbalances.^[4,5]^ Additionally, the requirement for regular, long-term dialysis sessions imposes significant physical and psychological burdens on patients, demanding high standards of care and professional expertise from healthcare providers.^[6]^ Optimizing the effectiveness of hemodialysis and improving patients’ quality of life remain critical clinical challenges.

### 1.3. Background and significance of the precision nursing model (PNM)

The PNM is an innovative nursing approach based on individualized and systematic care principles.^[7,8]^ With advancements in medical technology and increasing demands for chronic disease management, traditional nursing models have become insufficient to address the complex needs of DN patients undergoing hemodialysis. The PNM aims to provide more accurate and effective care through personalized care plans, enhanced multidisciplinary collaboration, and continuous quality improvement.^[7,8]^ Its core principle involves comprehensive assessment of patients’ medical conditions, lifestyles, psychological states, and other factors to develop tailored nursing interventions.^[7–9]^ Continuous monitoring and adjustments during the care process are integral for achieving optimal treatment outcomes and care quality.

Implementing the PNM in the care of DN patients undergoing hemodialysis can improve glycemic and blood pressure control, reduce the incidence of complications, and enhance patients’ adherence to treatment and quality of life through psychological support and health education.^[10–18]^ Studies have shown that the PNM significantly improves clinical indicators and patient satisfaction in DN care, demonstrating broad applicability.^[10–18]^ Therefore, in-depth research and promotion of this model in the context of hemodialysis for DN are crucial for advancing overall nursing standards and improving patient outcomes.

While this study focuses specifically on DN patients undergoing hemodialysis, the foundational principles of the PNM, including personalized care plans, multidisciplinary collaboration, and continuous quality improvement, suggest its potential relevance across diverse chronic conditions. Future studies should explore the adaptability of PNM in managing other high-risk populations requiring long-term care, tailoring interventions to address unique patient needs, healthcare system resources, and regional clinical practices. Such exploration may reveal modifications necessary for optimizing the model’s efficacy across varied healthcare settings.

## 2. Overview of DN and hemodialysis

### 2.1. Pathophysiological mechanisms of DN

DN is a progressive kidney disease resulting from long-term diabetes mellitus.^[19,20]^ The pathophysiology of DN is complex and involves hemodynamic and metabolic disturbances.^[21]^ Chronic hyperglycemia plays a pivotal role by inducing oxidative stress and forming advanced glycation end-products. These advanced glycation end-products activate various signaling pathways, including the protein kinase C and transforming growth factor-beta pathways, which contribute to glomerular hyperfiltration, hypertrophy, and ultimately glomerulosclerosis.^[22]^

Hyperglycemia also leads to the thickening of the glomerular basement membrane and mesangial matrix expansion, characteristic features of DN.^[23]^ Inflammatory processes and endothelial dysfunction further exacerbate renal damage.^[24]^ As the disease progresses, sustained hyperglycemia and hypertension accelerate renal function decline, manifesting as proteinuria, reduced glomerular filtration rate, and eventually, ESRD.^[25]^ Understanding these mechanisms is critical for developing targeted therapies to prevent or delay DN progression.

### 2.2. Principles and methods of hemodialysis

Hemodialysis is a key renal replacement therapy for patients with ESRD, including those with DN.^[26]^ Its primary function is to remove metabolic waste products and excess fluid from the bloodstream through diffusion and ultrafiltration.^[26]^ Blood is drawn from the patient’s vascular access and passed through a dialyzer, where it flows alongside a semipermeable membrane.^[26,27]^ On the other side of the membrane, a dialysate solution facilitates the removal of toxins, such as urea and creatinine, and helps regulate electrolyte balance.

Key components of the hemodialysis process include vascular access (usually an arteriovenous fistula, graft, or central venous catheter), the dialyzer (artificial kidney), and a dialysis machine that controls blood flow and dialysate delivery.^[26,27]^ Typically, patients undergo hemodialysis 3 times a week, with each session lasting approximately 4 hours. Despite its critical role, hemodialysis demands careful management to prevent complications such as infection, vascular access clotting, and hemodynamic instability.^[26]^

### 2.3. Application and challenges of hemodialysis in DN

The application of hemodialysis in DN patients presents unique challenges. Diabetic patients often have multiple comorbidities, such as cardiovascular disease and peripheral vascular disease, complicating hemodialysis management. Vascular access is particularly challenging due to the higher incidence of vascular calcification and poor vascular health in diabetic patients, leading to increased rates of access failure and infection.^[28]^

Cardiovascular complications are a major concern, as they are the leading cause of morbidity and mortality in this population. Managing fluid balance is crucial, as both fluid overload and dehydration can have severe consequences.^[29,30]^ Diabetic patients also frequently experience intradialytic hypotension, which can exacerbate renal and cardiac issues.^[31]^

Nutritional management is another critical aspect, requiring careful dietary planning to control blood glucose levels while adhering to dialysis-related dietary restrictions.^[32]^ The psychological burden of managing both diabetes and ESRD can reduce treatment adherence, emphasizing the need for comprehensive care strategies that address medical and psychosocial needs.^[33]^

In summary, hemodialysis is essential for DN patients with ESRD, but it requires a multidisciplinary approach to manage the complex challenges. Integrating PNM can enhance care by providing individualized and holistic management, improving patient outcomes and quality of life.

## 3. The concept and theoretical basis of the PNM

### 3.1. Definition of the PNM

The PNM is an innovative approach to patient care that emphasizes individualized and precise interventions tailored to the unique needs of each patient.^[7,8]^ This model is rooted in the principles of personalized medicine, which advocates for customizing healthcare based on individual characteristics such as genetic, environmental, and lifestyle factors.^[7,8]^ The primary goal of the PNM is to optimize clinical outcomes by integrating comprehensive patient assessments with targeted, evidence-based interventions.^[7–9]^ This approach involves continuous monitoring and adaptation of care plans to ensure they remain aligned with the patient’s evolving conditions and needs.

### 3.2. Theoretical basis and nursing philosophy of the PNM

The theoretical foundation of the PNM is grounded in several key concepts from nursing and healthcare sciences.^[7,34,35]^ Central to this model is a holistic view of the patient, which considers not only their physical health but also their psychological, social, and environmental contexts.^[9]^ This model is informed by patient-centered care theories that emphasize the importance of involving patients in their care decisions and tailoring interventions to their individual preferences and values.^[8,9]^

Furthermore, the PNM incorporates principles from systems theory, which acknowledges that patient outcomes are influenced by a complex interplay of factors and that effective care requires a coordinated, multidisciplinary approach.^[34,35]^ The model also leverages advancements in health informatics and data analytics to support the precise identification of patient needs and the development of targeted care strategies. The underlying philosophy of the PNM is one of the continuous improvement, where feedback from clinical outcomes is used to refine and enhance care protocols systematically.^[34,35]^

### 3.3. Current applications of the PNM in chronic disease management

The PNM has shown significant promise in the management of various chronic diseases, including diabetes, cardiovascular disease, and chronic kidney disease.^[36]^ In diabetes management, precision nursing involves comprehensive patient profiling to tailor dietary plans, medication regimens, and lifestyle interventions^[10–18]^ (Table 1). This personalized approach has been associated with improved glycemic control and a reduction in diabetes-related complications^[10–18]^ (Table 1).

**Table 1:** Summary of clinical studies on precision nursing model undergoing hemodialysis for diabetic nephropathy.

Study ref.	Condition	Treatment	Study type	Sample size	Findings
Zhao (2021)^[10]^	DNUH	PNM	RCT	100	Applying PNM to elderly DN patients on hemodialysis improves mental state, nutrition, and renal function.
Li (2020) ^[11]^	DNUH	PNM	RCT	62	For elderly DN patients on hemodialysis, PNM significantly improves psychological symptoms and reduces complications.
Zhang (2020)^[12]^	DNUH	PNM	RCT	102	PNM during hemodialysis for elderly DN patients alleviate anxiety and depression, reduce complications, and enhance satisfaction.
Zhou (2019)^[13]^	DNUH	PNM	RCT	98	PNM benefits DN patients on hemodialysis through significantly improving psychological well-being, alleviating negative emotions and reducing complications.
Jia (2019)^[14]^	DNUH	PNM	Retrospective study	60	Applying PNM to elderly DN patients undergoing hemodialysis optimizes treatment outcomes.
Lu (2019)^[15]^	DNUH	PNM	RCT	100	For elderly DN patients, PNM during hemodialysis significantly improve psychological well-being and ensure clinical safety.
Zhi (2018)^[16]^	DNUH	PNM	Retrospective study	80	Implementing PNM for elderly DN hemodialysis patients effectively increases care satisfaction and quality of life while reducing complications.
Li (2018)^[17]^	DNUH	PNM	RCT	100	Implementing PNM for elderly DN hemodialysis patients clinically effectively alleviates negative emotions and reduces complications.
Xie (2017)^[18]^	DNUH	PNM	RCT	90	PNM alleviates negative emotions, reduces anxiety and depression, lowers hemodialysis complications, and boosts patient satisfaction with nursing care.

DN = diabetic nephropathy, DNUH = diabetic nephropathy undergoing hemodialysis, PNM = precision nursing model, RCT = randomized controlled trial.

For cardiovascular disease, precision nursing tailors interventions based on individual risk factors, leading to better blood pressure management and a reduction in cardiovascular events.^[37]^ In chronic kidney disease management, the PNM facilitates the creation of individualized care plans that address the diverse needs of patients, including dietary management, fluid balance, medication adherence, and psychosocial support.^[11–13,15–18]^ By continuously monitoring patient responses and adjusting care plans accordingly, precision nursing helps to stabilize kidney function, delay disease progression, and enhance overall quality of life.^[11,15,16]^

Empirical studies have demonstrated that the PNM not only improves clinical outcomes but also enhances patient satisfaction and reduces healthcare costs by preventing complications and minimizing hospital readmissions.^[12,15,16,18]^ The integration of technology, such as electronic health records and telehealth platforms, supports the efficient implementation of precision nursing by providing real-time data and facilitating communication among healthcare providers.

In summary, the PNM represents a significant advancement in chronic disease management. Its focus on individualized care, supported by a robust theoretical foundation and modern technological advancements, aligns well with the current trends towards personalized medicine and value-based healthcare. As this model continues to evolve, it holds great potential to further improve patient outcomes and transform the landscape of chronic disease management.

## 4. Application of the PNM in hemodialysis for patients with DN

### 4.1. Implementation steps and strategies of the PNM

Given the variability in healthcare environments, these implementation strategies could be further refined and adapted to suit other chronic disease settings and diverse patient demographics. Modifying specific elements, such as interdisciplinary collaboration frameworks or intervention protocols, to address condition-specific requirements and resource availability could enhance the PNM’s broader applicability, promoting individualized, high-quality care across various patient populations.^[38]^

#### 4.1.1. Development of individualized care plans

At the foundation of the PNM lies the development of personalized care plans tailored to each patient’s unique needs. These plans begin with an in-depth assessment to identify each patient’s specific risk factors, preferences, and potential obstacles to treatment adherence.^[10,12]^ Care plans are meticulously crafted to manage critical aspects such as glycemic control, fluid balance, and overall wellness, adapting dynamically to the patient’s evolving health status.^[13,16]^

#### 4.1.2. Multidisciplinary team collaboration and communication

The model thrives on the coordinated efforts of a multidisciplinary team, including nephrologists, endocrinologists, dietitians, and other healthcare specialists.^[14,15]^ This team approach ensures comprehensive care by holding regular interdisciplinary meetings, which facilitate effective communication and the integration of diverse therapeutic perspectives to create holistic treatment strategies.^[11,17]^

#### 4.1.3. Training and assessment of nursing staff

Nursing staff are crucial to the successful implementation of precision care. Specialized training programs are essential for nurses to deepen their understanding of diabetes management, hemodialysis techniques, and patient-centered care principles.^[18]^ Ongoing education and regular skill assessments help maintain high standards of care and adherence to updated clinical practices.^[10,12]^

Despite these efforts, real-world implementation of the PNM is often hindered by practical challenges, such as limited resources and the high demand for specialized training.^[39]^ Healthcare facilities may struggle to retain adequate staffing levels and provide continuous training, both essential for the sustained success of PNM.^[40]^ To address these issues, healthcare organizations might consider scalable and adaptable training programs alongside strategic resource allocation to enhance the feasibility of PNM in diverse clinical settings.

### 4.2. Specific intervention measures of the PNM

#### 4.2.1. Blood glucose control and dietary management

Collaboration with dietitians is vital for constructing individualized dietary plans that stabilize glucose levels and meet the nutritional demands and restrictions of hemodialysis.^[10,14]^ Continuous monitoring and timely adjustments of dietary and medication plans are imperative to manage glycemic levels effectively during dialysis treatments.^[15,17]^

#### 4.2.2. Monitoring and adjustment during dialysis sessions

Constant monitoring of vital parameters and biochemical indices during dialysis sessions is crucial. It enables nurses to promptly identify and manage potential complications such as intradialytic hypotension, electrolyte imbalances, or issues with vascular access.^[16,18]^ Immediate adjustments to dialysis settings, including ultrafiltration rates and dialysate composition, are coordinated with nephrologists to ensure optimal treatment outcomes.^[11,13]^

#### 4.2.3. Psychological support and patient education

Psychological support and comprehensive patient education form integral components of the precision nursing care framework. Nurses provide emotional support and practical coping strategies to assist patients in managing the psychological burdens of chronic illness and treatment regimens.^[12,14]^ Educational initiatives focus on empowering patients to take an active role in their own care, thereby enhancing their ability to manage medications, recognize symptoms, and implement necessary lifestyle changes.^[17,18]^

In summary, the PNM embodies a detailed, patient-focused approach to managing hemodialysis in DN patients. By individualizing care strategies, ensuring collaborative practice among healthcare professionals, and enacting targeted interventions, this model aims to optimize therapeutic outcomes, heighten patient satisfaction, and significantly improve quality of life for this vulnerable population.

## 5. Evaluation of the effectiveness of the PNM in hemodialysis for DN

### 5.1. Clinical efficacy evaluation indicators

#### 5.1.1. Improvement in clinical parameters

The PNM’s effectiveness is prominently assessed through significant improvements in glycemic control and renal function parameters. Personalized management strategies like tailored dietary guidance and vigilant glycemic monitoring are fundamental. Effective management is indicated by reductions in hemoglobin A1c, stabilization of serum creatinine levels, and decreased urinary albumin excretion rates, demonstrating improved renal health and management efficacy^[10,12,14]^ (Table 1).

#### 5.1.2. Incidence of complications

The model’s impact on reducing complications associated with DN and hemodialysis is crucial. By optimizing dialysis parameters and fluid management, precision nursing aims to minimize occurrences of cardiovascular incidents, electrolyte imbalances, and dialysis access infections. A reduction in these complications indicates a safer, more effective treatment regimen, enhancing patient outcomes^[13,15,17]^ (Table 1).

#### 5.1.3. Patient quality of life

Quality of life improvements provide a holistic view of the PNM’s impact on patients. This model not only manages medical needs but also supports patients’ psychosocial aspects, enhancing emotional and social well-being. Measurable improvements in areas such as physical functioning and emotional health reflect the model’s overall benefits, indicating successful integration of care^[16,18]^ (Table 1).

### 5.2. Nursing quality evaluation

#### 5.2.1. Nursing satisfaction

Patient perceptions of nursing care quality are integral for evaluating the PNM. High levels of patient satisfaction, reflecting positive experiences, and effective communication during hemodialysis sessions, are indicative of the model’s success. Satisfied patients are more likely to adhere to treatment plans, positively impacting therapeutic relationships and treatment outcomes^[10,14]^ (Table 1).

#### 5.2.2. Compliance during nursing process

Compliance with the nursing process under the PNM is critical for ensuring effective treatment outcomes. This model stresses the importance of patient education and empowerment, promoting adherence to prescribed treatment, and lifestyle adjustments. Monitoring compliance helps identify successful engagements and areas requiring more focused interventions.^[11,15]^

#### 5.2.3. Professional competence enhancement of nursing staff

The development of nursing staff’s professional competence is essential for delivering optimal care. The PNM supports continuous professional development through regular training and performance evaluations. These assessments help ensure that nursing staff are competent, confident, and capable of delivering high-quality, evidence-based care, thereby enhancing the overall treatment efficacy.^[12,13,16]^

In summary, a rigorous and comprehensive evaluation of the PNM in hemodialysis for DN is conducted across several domains: clinical outcomes, patient satisfaction, and nursing quality. This structured evaluation not only highlights areas for improvement but also aids in refining care delivery processes to boost the overall quality of care for patients with DN.

## 6. Challenges and strategies in implementing the PNM

### 6.1. Barriers in PNM implementation

To extend PNM’s utility beyond DN patients undergoing hemodialysis, future work should examine how specific implementation challenges, including resource limitations and staff training, may differ across healthcare contexts. Investigating these challenges could provide insights into required adjustments, such as simplified intervention protocols or customized training programs, thereby supporting PNM’s effective integration in settings with varied resource levels and patient demographics.^[41]^

A major obstacle to PNM implementation in routine practice is the limitation of resources, including financial support, staffing capacity, and facility infrastructure.^[42,43]^ These constraints can restrict the ability of healthcare teams to deliver personalized and consistent care, potentially impacting patient outcomes. Furthermore, PNM requires a level of staff training and expertise that can be challenging to maintain without structured institutional support.^[44]^ By optimizing resource allocation and prioritizing targeted funding for precision nursing, healthcare organizations can facilitate more effective integration of PNM into daily clinical practice.^[43]^

#### 6.1.1. Inadequate nursing resources

A primary challenge in implementing the PNM is the scarcity of nursing resources, both in terms of qualified staff and time constraints.^[41,45]^ The model demands dedicated nursing personnel with specialized training in diabetes management and hemodialysis. However, understaffing and heavy workloads prevalent in healthcare facilities hinder the delivery of personalized care, potentially compromising patient outcomes.

#### 6.1.2. Patient compliance issues

Ensuring patient compliance with prescribed treatment regimens and nursing interventions presents another significant obstacle.^[46,47]^ Despite receiving personalized care plans, some DN patients may struggle with adherence due to socioeconomic constraints, health literacy barriers, or psychological resistance to lifestyle modifications.^[46,47]^ Nonadherence undermines the effectiveness of precision nursing interventions and impedes disease management efforts.

#### 6.1.3. Difficulty in multidisciplinary collaboration

Effective implementation of the PNM hinges on seamless collaboration among multidisciplinary healthcare teams.^[48]^ However, coordinating care across different specialties, including nephrology, endocrinology, nutrition, and psychology, can be challenging.^[48,49]^ Communication barriers, conflicting priorities, and limited interdisciplinary training may hinder teamwork and compromise integrated care delivery to DN patients undergoing hemodialysis.^[48,49]^

### 6.2. Solutions and future directions

#### 6.2.1. Strengthening nursing personnel training and management

Addressing the shortage of nursing resources requires prioritized investment in training and retaining qualified nursing staff.^[50]^ Continuous education programs focusing on diabetes management, hemodialysis procedures, and patient-centered care principles can enhance nurses’ competencies in delivering precision nursing interventions.^[51]^ Additionally, supportive management practices, such as workload optimization and recognition of nursing achievements, improve staff morale and retention rates.

While structured training programs are essential to PNM, healthcare institutions may face practical limitations, such as budgetary constraints and access to training resources, which can impede the establishment of continuous education protocols.^[52]^ Especially in resource-limited settings, maintaining the standard of training required for precision nursing can be challenging.^[52]^ Developing cost-effective, modular training solutions and fostering collaborations with educational institutions could provide more sustainable pathways for PNM training, thus enhancing its accessibility and long-term viability in clinical settings.

#### 6.2.2. Optimization of dialysis center management models

Optimizing dialysis center management models facilitates the implementation of the PNM.^[53]^ This includes streamlining care processes, improving workflow efficiency, and leveraging technology to automate administrative tasks. Allocating dedicated time for multidisciplinary team meetings and care coordination sessions fosters collaboration and enhances communication among healthcare providers. Furthermore, adopting quality improvement initiatives and benchmarking best practices drive continuous performance enhancement in dialysis center management.^[54]^

#### 6.2.3. Integration of technological solutions

Technological advancements, such as remote monitoring systems, augment the delivery of precision nursing care.^[55]^ Remote monitoring enables real-time tracking of patient vital signs, treatment adherence, and clinical outcomes outside traditional healthcare settings.^[55]^ Providing timely feedback and early intervention opportunities, remote monitoring enhances patient engagement, improves self-management behaviors, and facilitates proactive disease management. Integrating telehealth platforms and mobile applications into the PNM promotes continuity of care and expands access to specialized support services for DN patients undergoing hemodialysis.^[56]^

In summary, overcoming challenges in implementing the PNM requires a multifaceted approach encompassing workforce development, process optimization, and technological innovation. By strengthening nursing personnel training, optimizing dialysis center management, and leveraging technological solutions, healthcare organizations can advance personalized care delivery to DN patients undergoing hemodialysis, ultimately improving clinical outcomes and enhancing patient experience.

## 7. Summary and future outlook

### 7.1. Significance of PNM in hemodialysis for DN

The PNM emerges as a critical approach in enhancing care and outcomes for DN patients undergoing hemodialysis. Through tailored interventions and interdisciplinary collaboration, precision nursing addresses the intricate challenges of managing concurrent diabetes and renal disease. By devising personalized care plans, diligently monitoring patients, and imparting education, this model not only improves glycemic control but also preserves renal function and enhances overall well-being. Its foundation in evidence-based practice and continuous quality enhancement ensures optimal patient outcomes and satisfaction.

### 7.2. Future research directions and application prospects

Future research in precision nursing for DN patients undergoing hemodialysis should prioritize several key avenues. Firstly, longitudinal investigations into the enduring effects of precision nursing interventions on clinical outcomes and quality of life are necessary. Exploring innovative technological solutions, such as artificial intelligence and wearable devices, to augment remote monitoring and self-management capabilities holds promise. Additionally, conducting comprehensive cost-effectiveness analyses and health economic evaluations will offer insights into the scalability and sustainability of PNMs in real-world clinical settings. Overall, the ongoing advancement and integration of precision nursing approaches present exciting prospects for enhancing care and well-being among DN patients undergoing hemodialysis, ultimately contributing to superior health outcomes and healthcare delivery.

## Author contributions

**Conceptualization:** Jing Ma, Dan-Wei Ma.

**Data curation:** Jing Ma, Dan-Wei Ma.

**Investigation:** Dan-Wei Ma.

**Methodology:** Jing Ma, Dan-Wei Ma.

**Project administration:** Dan-Wei Ma.

**Resources:** Jing Ma, Dan-Wei Ma.

**Supervision:** Dan-Wei Ma.

**Validation:** Jing Ma, Dan-Wei Ma.

**Visualization:** Jing Ma, Dan-Wei Ma.

**Writing – original draft:** Jing Ma, Dan-Wei Ma.

**Writing – review & editing:** Jing Ma, Dan-Wei Ma.
